# A Case of Giant Cell Arteritis Presenting As Catastrophic Posterior Circulation Stroke: A Diagnostic Dilemma

**DOI:** 10.7759/cureus.27961

**Published:** 2022-08-13

**Authors:** Joshua Wong, Siang Chan, Ashit Shetty

**Affiliations:** 1 Stroke Service, Nottingham University Hospitals NHS Trust, Nottingham, GBR

**Keywords:** glucocorticoids, cerebrovascular accidents, doppler ultrasonography, echocardiogram (echo), contrast-enhanced imaging, coronary thrombus, posterior circulation stroke, temporal artertitis, giant cell arteritis with polymyalgia rheumatica

## Abstract

Giant cell arteritis (GCA) is an immune-mediated systemic vasculitis usually seen in the older population. We describe a case of a 75-year-old woman who presented with jaw claudication and temporal headache. A colour duplex ultrasonography and later biopsy of the temporal arteries confirmed GCA and she was commenced on oral steroids. She was subsequently readmitted with a new worsening vision of both eyes and confusion. Her brain images revealed acute bilateral vertebral artery thrombus with haemorrhagic transformation. She was loaded on intravenous steroids. The next day she developed vomiting, bilateral visual loss and a cardiac arrest from ventricular fibrillation. Following the return of spontaneous circulation, she was taken to the cardiac catheterisation laboratory for a coronary angiogram, which showed diffuse thrombus at the apical left anterior descending artery. A bedside echocardiogram revealed a sizable left ventricular thrombus. She was managed with heparin and antiplatelet therapy.

This case presented a complex diagnostic dilemma to the medical team as vasculitis, atherosclerosis, and cardiac emboli could have contributed to her stroke and visual loss. This patient also had some vascular risk factors for occlusive cerebrovascular disease, potentially suggesting a clinical event with multiple aetiologies.

Stroke and visual loss are rare but serious complications of GCA, which require a high index of suspicion and early treatment with corticosteroids to improve prognosis. Although a temporal artery biopsy remains to be the definitive diagnostic modality for GCA, the use of radiological investigations in the diagnosis of GCA is increasingly common. A non-invasive colour duplex ultrasonography of the temporary arteries could be used to assess GCA in highly suspected patients. Echocardiograms and contrast-enhanced body imaging should be performed in patients with suspected or established GCA to assess for secondary thromboembolic and vascular complications.

## Introduction

Giant cell arteritis (GCA) is an immune-mediated, systemic vasculitis of the medium to large-sized arteries, predominantly seen in the older population over the age of 50. GCA is the most common form of primary vasculitis in Western Europe [1]. Women are two times more likely to be affected by GCA than men [2].

The clinical manifestations of GCA are classified into intracranial and extracranial features. In addition, most patients with GCA may have one or more non-specific, constitutional symptoms including fever, lethargy, malaise, weight loss, and anorexia [2]. Up to 90% of patients with GCA have classical intracranial symptoms, which include new temporal headache, scalp tenderness (e.g., pain when brushing hair), jaw claudication, and transient or permanent vision loss [3]. Although less frequently encountered, extracranial manifestations of GCA are also reported. Neurological symptoms such as mononeuropathy, peripheral neuropathy, and myelopathy are experienced in some patients with GCA. Up to 10% of patients with GCA may have respiratory tract symptoms such as cough, sore throat, and hoarseness [2].

GCA is commonly associated with polymyalgia rheumatica (PMR), which presents as pain and morning stiffness in specific muscle groups, predominantly around the neck, shoulders, upper arms, and pelvic girdle [4]. Both GCA and PMR can co-exist or they can occur separately.

GCA is a medical emergency and patients with suspected GCA must be diagnosed and treated immediately to prevent irreversible consequences of large vessel vasculitis. There are some published case reports on patients suffering from rare complications of GCA such as bilateral visual loss, stroke, and coronary events. None of these however describe a case of co-existing cerebral-coronary manifestations, which presents a diagnostic dilemma that requires complex decision-making from the multi-disciplinary team. We present an exceptional case of a 75-year-old woman who was found to have bilateral visual loss and catastrophic vertebrobasilar stroke and coronary thrombus shortly after a diagnosis of GCA. Could these manifestations be solely attributed to her underlying GCA?

## Case presentation

Our patient was a 75-year-old, right-handed, Caucasian woman who presented to the emergency room with a three-month history of intermittent jaw claudication and right-sided temporal headaches, and a three-week history of blurry vision in the right eye. She had a past medical history of hypertension, transient ischaemic attack (TIA) in 2016, osteoarthritis, vertigo, rotator cuff injury to the right shoulder, and anxiety disorder. Her past surgical history was significant for a hysterectomy for fibroids in 2006. Her medications on presentation included Paracetamol 1 gram (g) twice daily, Amlodipine 5 milligram (mg) once daily, Atorvastatin 20 mg once daily, Bendroflumethiazide 2.5 mg once daily, Behahistine 8 mg twice daily, Clopidogrel 75 mg once daily, Dosulepin 25 mg once daily, Lorazepam 0.5 mg once daily and Fenbid Ibuprofen gel as required. She lived alone in a house with stairs and was independent with all her activities of daily living. She had been smoking 15 cigarettes a day for about 45 years. She denied any alcohol consumption.

On examination, a right optic disc swelling was elicited. Systemic examinations were otherwise normal. Her laboratory results are shown in Table 1. Significant results include mildly elevated C-reactive protein (CRP) and erythrocyte sedimentation rate (ESR). Her initial computed tomography (CT) head scan revealed no acute findings.

**Table 1:** Laboratory results

Laboratory Results (SI units; conversion units)	First Presentation	First Admission	Second Admission	Normal Range
Haemoglobin (g/dL)	12.5	12.9	13.2	(11.5 - 16.5)
White blood cells (10^9^/L)	11.12	11.60	22.59	(3.6 - 11.0)
Platelet count (10^9^/L)	425	434	360	(140 - 400)
Sodium (mmol/L)	134	136	127	(133 - 146)
Potassium (mmol/L)	3.2	3.0	3.6	(3.5 - 5.3)
Urea (mmol/L)	6.8	8.3	7.8	(2.5 - 7.8)
Creatinine (μmol/ L)	75	84	58	(45 - 84)
eGFR	67	59	87	(> 90)
C-reactive protein (CRP) (mg/ L)	27	23	20	(< 5)
Erythrocyte sedimentation rate (ESR) (mm/hour)	28	17	16	(5 - 15)

In view of the long duration (three months) and almost completely resolved symptoms during the outpatient consultation, she was discharged with eye lubricants and safety netting advice. Nevertheless, she was reviewed at the rheumatology clinic 10 days after and a colour duplex ultrasonography of her bilateral temporal arteries suggested GCA. She was started on high-dose oral prednisolone 60 mg once daily with a proton pump inhibitor and calcium and vitamin D supplementation for gastro- and bone protection, respectively.

**Figure 1:**
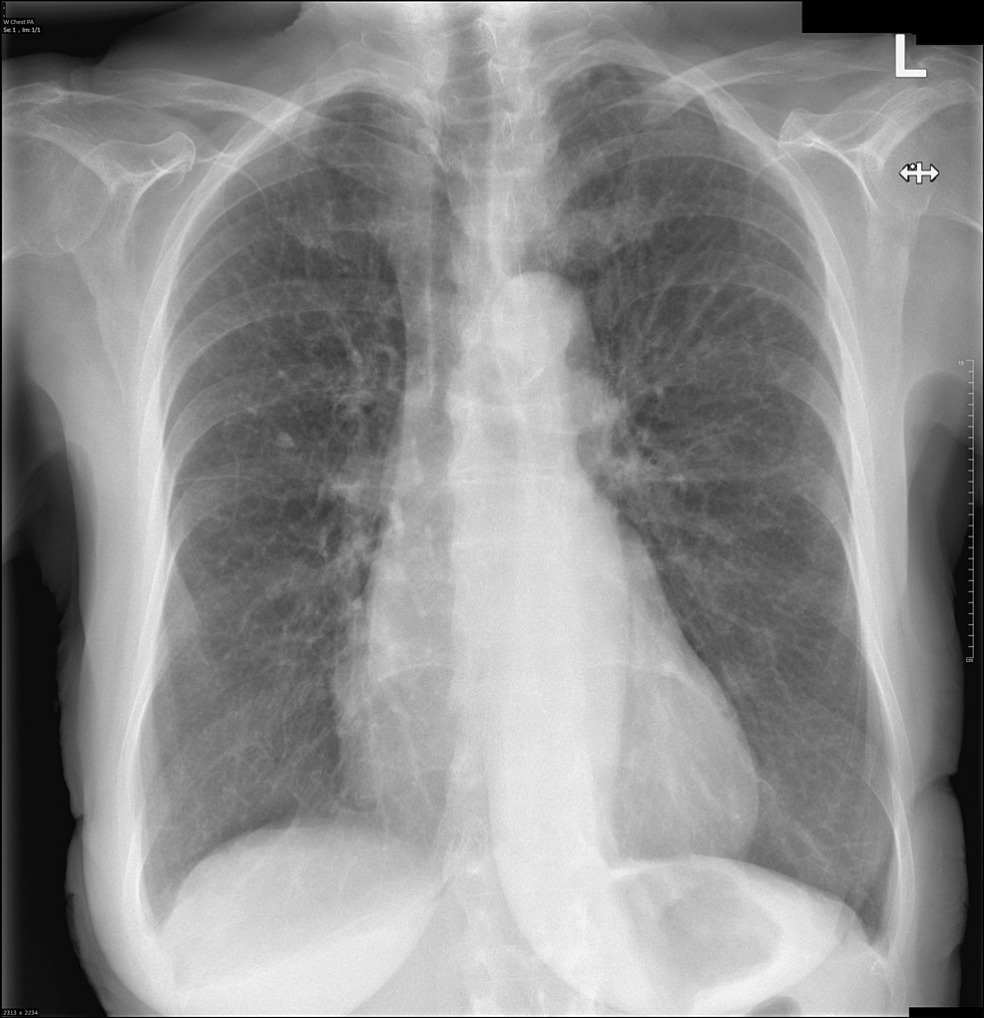
A posterior-anterior view of a chest radiograph. The heart is enlarged with a cardiothoracic ratio of 54%. The lungs are hyperinflated. No focal lung lesion, consolidation, or pleural effusions are identified.

A routine chest radiograph (Figure 1) was performed prior to initiating treatment. It revealed an incidental finding of cardiomegaly with a cardiothoracic ratio of 54%.

A day later, she presented to the hospital with new-onset, worsening vision of both eyes, vomiting, and confusion. On examination, a right pronator drift and right homonymous hemianopia were elicited. Her bilateral visual acuity was recorded to be hand movements on arrival. She was afebrile and the remaining of her vital signs were otherwise normal. As seen in Table 1, it is worth noting that both her CRP (17, was 28) and ESR (23, was 27) were improving at that time. An extended vasculitis workup was only positive for anti-nuclear antibodies (ANA), and the rest of the autoimmune screen was unremarkable. In view of her mildly raised white cell counts and inflammatory markers, she was started on broad-spectrum antibiotics to prophylactically treat community-acquired and aspiration pneumonia.

**Figure 2:**
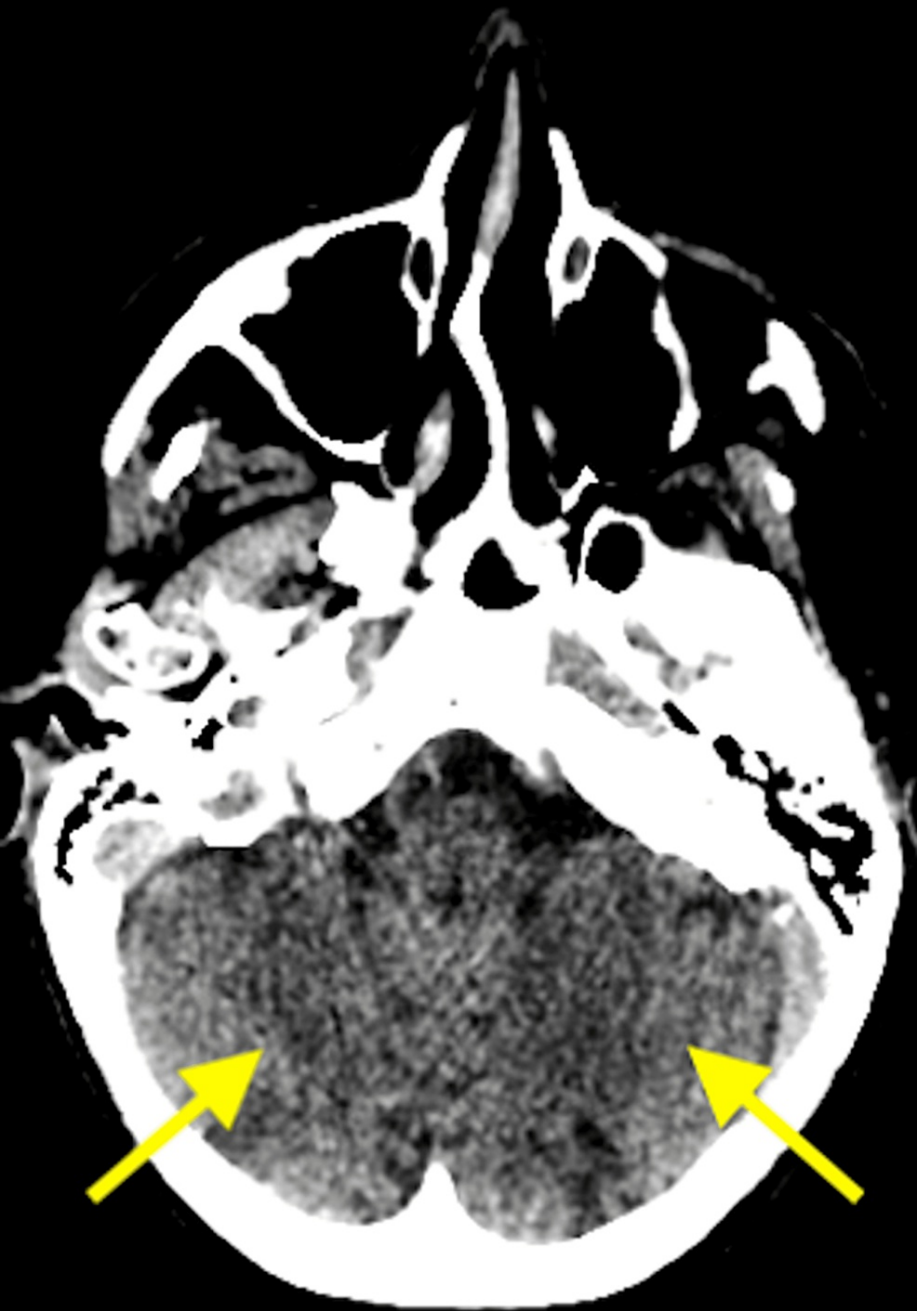
An axial CT head scan showing acute bilateral superior cerebellar infarcts (arrows).

**Figure 3:**
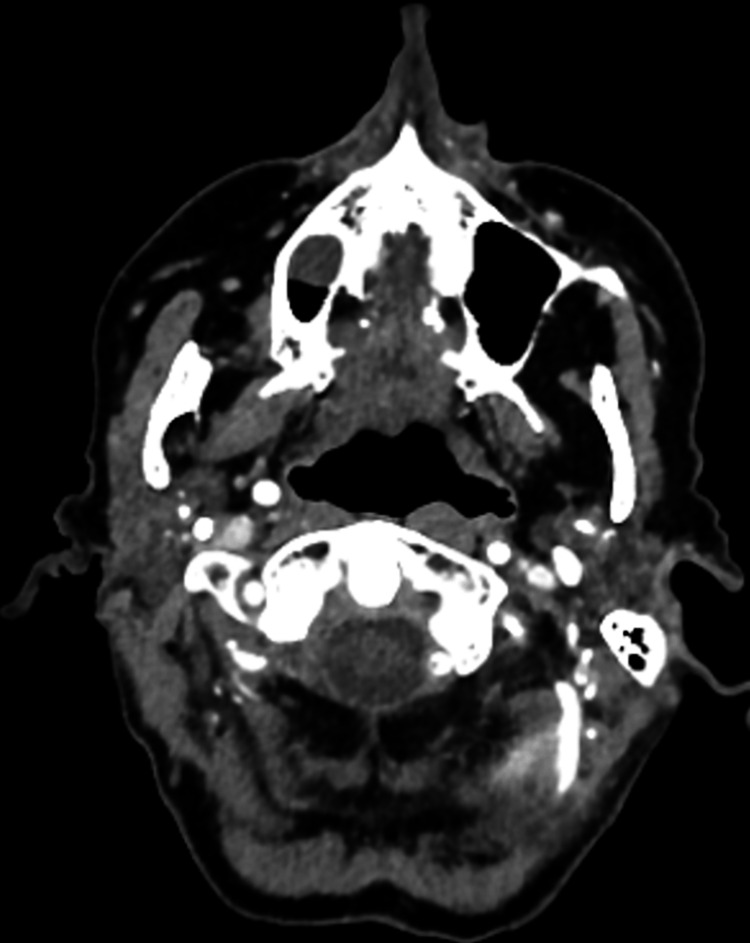
An axial CT head angiogram demonstrating thrombus in both distal vertebral arteries.

A CT head scan and angiogram (Figures 2, 3) were performed, which revealed acute bilateral superior cerebellar infarcts with thrombus in both distal vertebral arteries.

**Figure 4:**
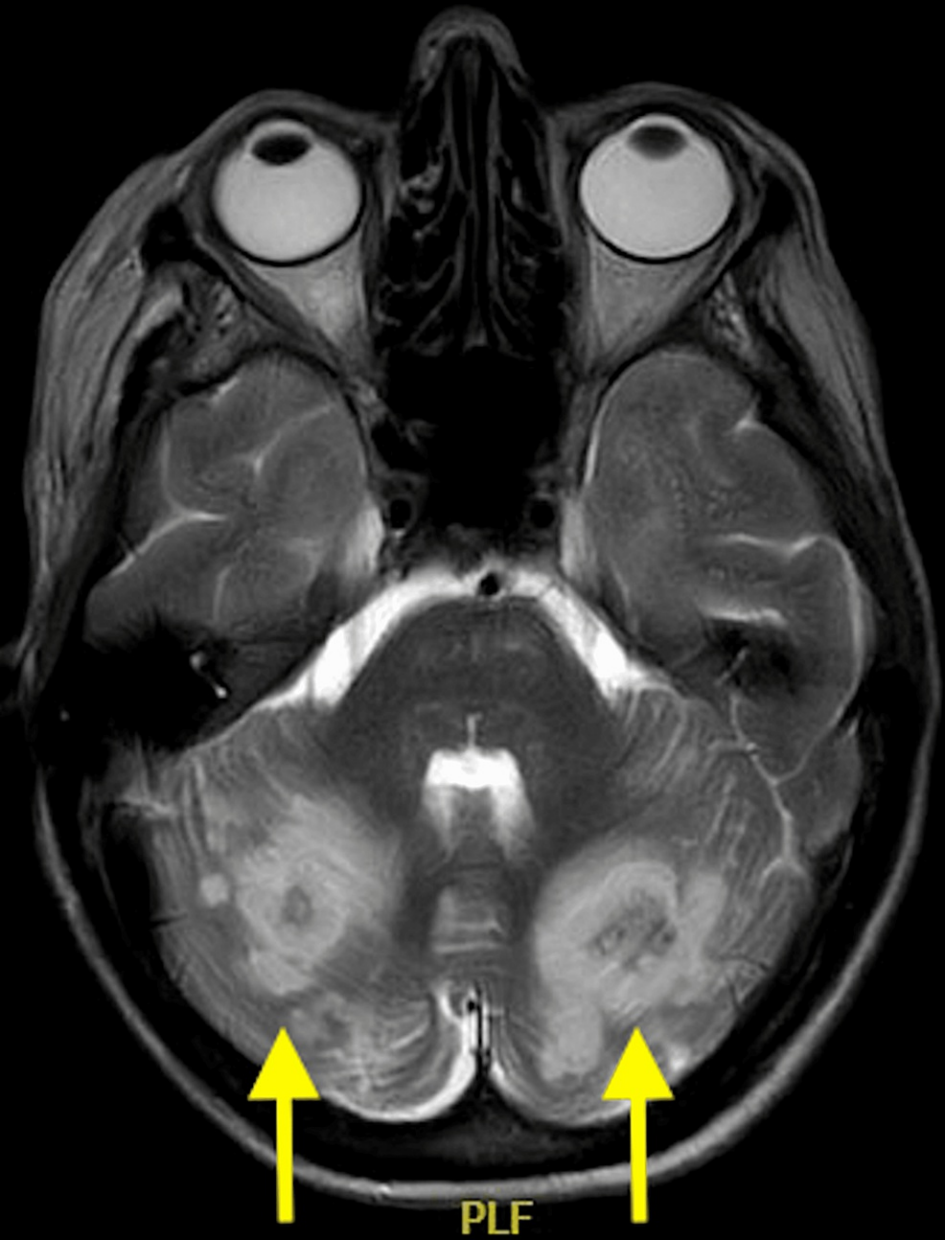
An axial, T2 magnetic resonance image showing extensive bilateral posterior cerebral artery territory infarcts complicated by haemorrhagic transformation (arrows).

**Figure 5:**
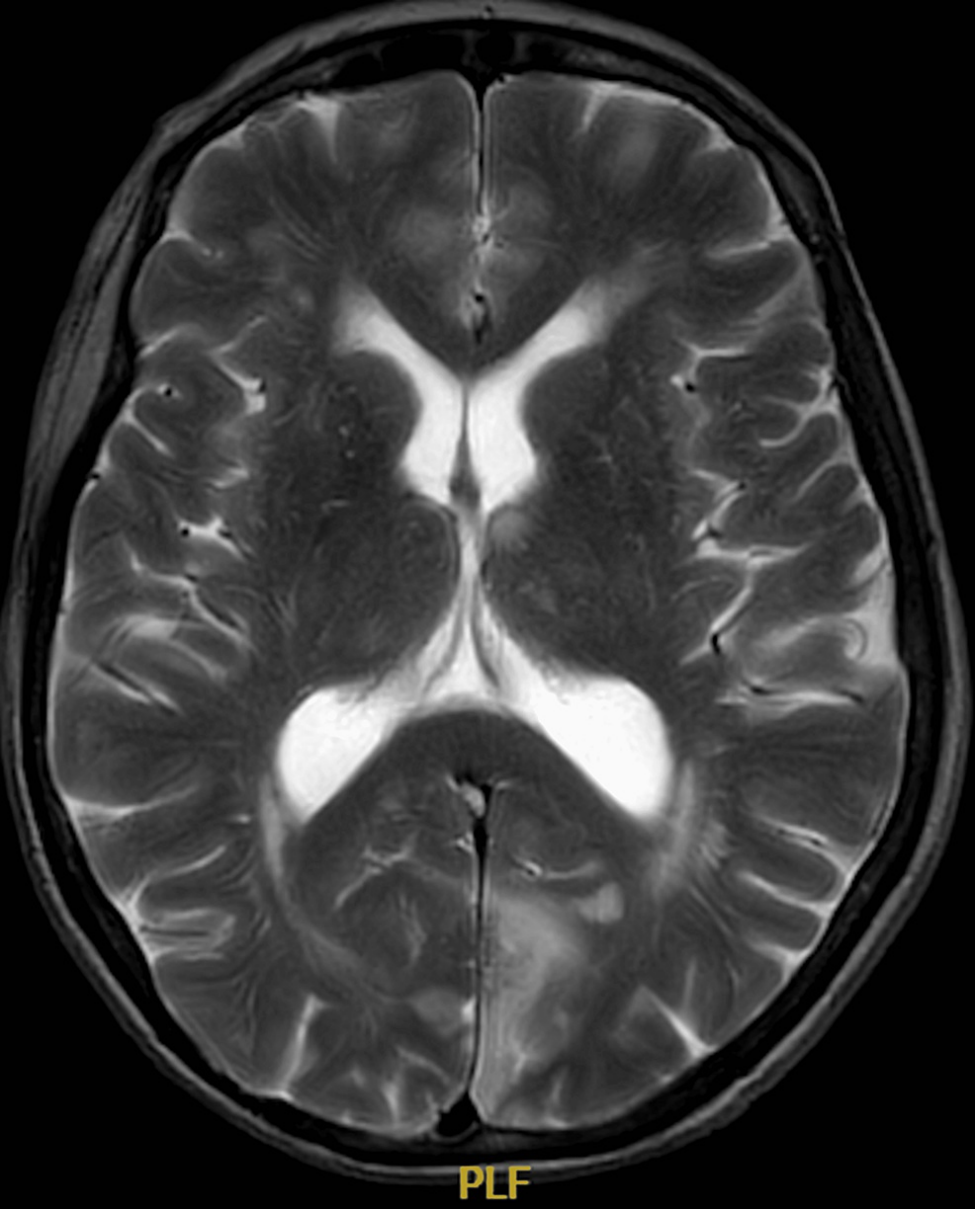
An axial, T2 magnetic resonance image showing extensive bilateral posterior cerebral artery territory infarcts involving the medial occipital lobes and the basal ganglia and thalamus region.

**Figure 6:**
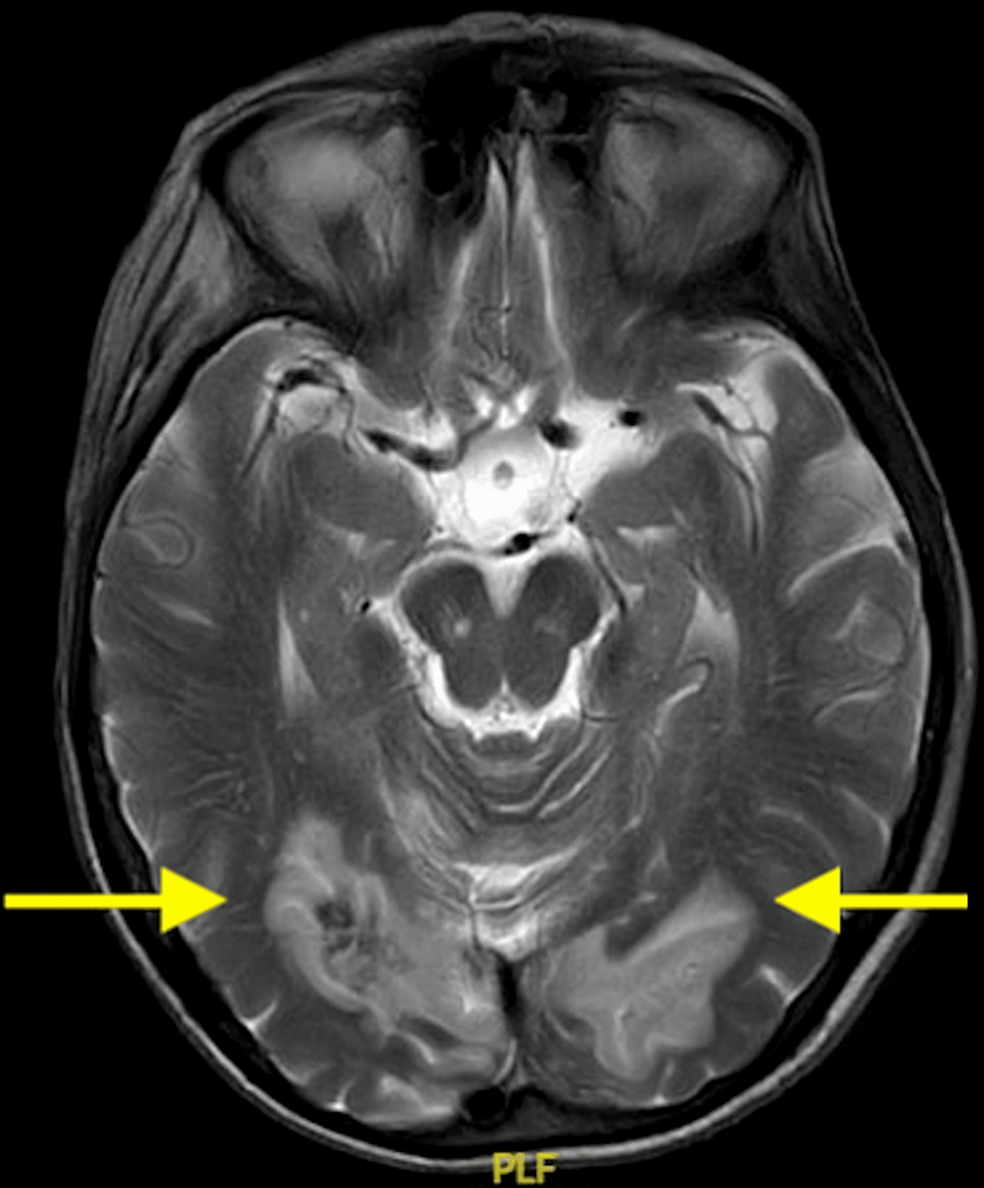
An axial, T2 magnetic resonance image showing extensive bilateral posterior cerebral artery territory infarcts involving the cerebellar hemispheres and medial occipital lobes. This is complicated by haemorrhagic transformation in the bilateral cerebellar hemisphere infarcts (arrows).

She went on to have an inpatient magnetic resonance (MRI) head scan, which revealed extensive haemorrhagic changes in the bilateral posterior territory infarcts (Figures 4-6).

 

It was believed that her stroke was secondary to GCA, she was started on intravenous methylprednisolone for three days and daily loading-dose aspirin. The results of her temporal artery biopsies came back on her third day of admission, confirming GCA with transmural inflammation and the presence of multinucleated giant cells in the arterial wall. Nonetheless, she continued to improve clinically at the stroke unit. Her vision had improved remarkably, although a residual bilateral inferior quadrantanopia with macular sparring was found on examination. She was discharged five days later and continued on daily 75 mg of clopidogrel and 60 mg of prednisolone.

**Figure 7:**
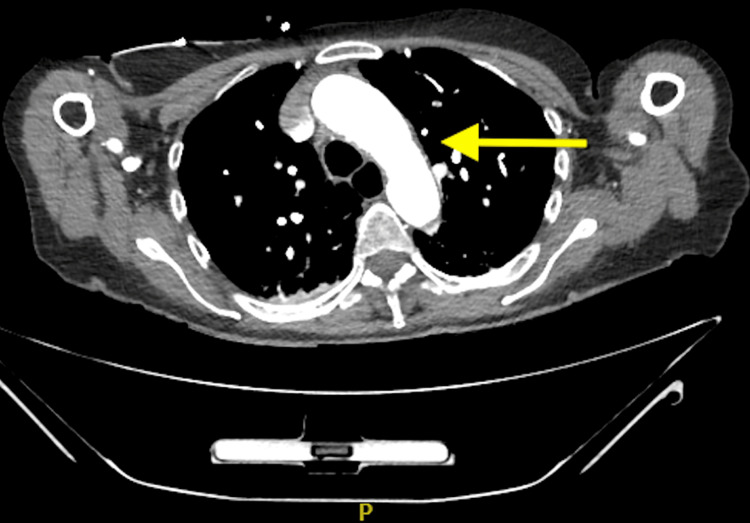
An axial, contrast-enhanced CT image of the thoracic aorta revealed no evidence of aortic dissection and aneurysm.

**Figure 8:**
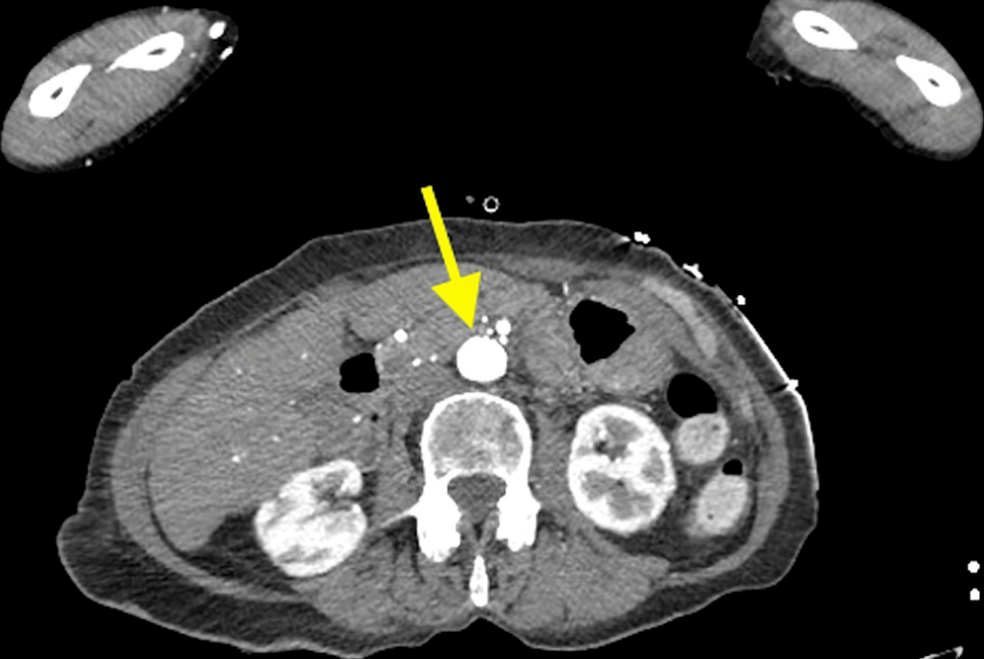
An axial, contrast-enhanced CT image of the abdominal aorta revealed no evidence of aortic dissection and aneurysm. Note a normal calibre aorta with no wall thickening or peri-aortic inflammatory stranding (arrows).

She was readmitted the next day with headache, vomiting, and representation of bilateral visual loss, to the perception of light only. While waiting to be seen, she suffered from a cardiac arrest secondary to ventricular fibrillation. Following a return of spontaneous circulation, an electrocardiogram was taken, which demonstrated anterolateral ST-segment elevation. She was taken to the cardiac catheterisation laboratory for a coronary angiogram, which showed no significant stenosis of coronary arteries but diffuse thrombus at the apical left anterior descending artery. She was later transferred to the intensive care unit, where she was intubated and ventilated, and given vasopressors. A bedside echocardiogram revealed a sizable left ventricular thrombus and severe left ventricle impairment (ejection fraction < 35%). The decision to treat the coronary thrombus was difficult given a recent haemorrhagic transformation of cerebral infarcts in this patient with established GCA. She was medically managed with intravenous heparin followed by antiplatelet therapy. Her steroids were switched to tocilizumab given her heart failure. Contrast-enhanced CT scans of the head, neck, thorax, abdomen, and pelvis were performed (Figures 7, 8), which did not reveal any evidence of new or progressive infarct, systemic vasculitis, or aortic or carotid dissection and aneurysm.

On the same night, there were ongoing concerns regarding her dilated and unreactive pupils bilaterally. A repeat CT head and angiogram revealed stable appearances of the established posterior circulatory infarct. In view of her ongoing unresponsiveness, apnoea on trial without ventilation, anuria, and deteriorating blood parameters indicative of multiple organ failures, she was withdrawn from life-sustaining treatment two days after and she passed away peacefully.

## Discussion

This patient presented with a sub-acute onset of classical GCA symptoms including new temporal headache, mandibular claudication, and blurry vision. New-onset headaches or gradually worsening pre-existing headaches in an elderly patient should always raise an alarm about the possibility of GCA or intracranial pathologies. Headache and jaw claudication is understood to be the results of inflammatory vasculitis of the temporary artery and maxillary artery respectively. Hypoperfusion of the mastication muscles causes discomfort and pain when chewing and talking, leading to jaw claudication [2].

Our patient had mildly elevated inflammatory markers including CRP and ESR, which declined steadily with the administration of prednisolone. Serial inflammatory markers are important in establishing a diagnosis as well as monitoring treatment response. The presence of persistent systemic inflammation releases cytokines such as Interleukin 6 (IL-6), which induces the hepatic synthesis of acute-phase protein CRP [5]. The marked increase of acute phase reactants including CRP and ESR is the hallmark of GCA. However, a high serum ESR level could be associated with advancing age and the presence of other comorbidities such as malignancy, chronic kidney disease, and anaemia. Thus, CRP is considered a more sensitive marker than ESR in diagnosing GCA. It is worth noting, however, that normal CRP and ESR levels do not rule out the diagnosis of GCA as a small group of patients with GCA may have normal inflammatory markers at diagnosis [6].

The definitive diagnostic test for GCA remains to be a temporal artery biopsy. It should be considered when there is a clinical suspicion of GCA. Nonetheless, treatment with corticosteroids should not be delayed with this procedure. Furthermore, this procedure may be limited by a high false negative rate due to the presence of skip lesions, which are not uncommon. A large specimen may be required occasionally to increase the diagnostic yield, which in turn increases the risk of potential intraoperative bleeding.

The established complications and high false negative rate of a temporary artery biopsy have led to the increased utilisation of radiological investigations in the diagnosis of GCA. A non-invasive duplex ultrasound scan is the first-line imaging modality for suspected GCA, given its availability and lack of radiation exposure. The presence of a hypoechoic wall thickening - “halo sign” around both temporal arteries represents inflammatory vessel wall edema and is suggestive of temporal artery inflammation [7]. The sensitivity and specificity of ultrasound diagnosis of GCA were reported to be 91.6% and 95.8%, respectively, when compared with the histological diagnosis in a study of 451 consecutive patients with GCA [8].

The use of computed tomography angiography (CTA) and magnetic resonance angiography (MRA) has gained popularity in the evaluation of vasculitis in patients with suspected GCA, demonstrating promising diagnostic performance, especially in medium and large-sized arteries such as the abdominal and thoracic aorta, and head and neck arteries and their branches. Common radiological findings of GCA are vessel wall thickening and systemic or localised contrast enhancement of the arterial wall secondary to mural inflammation [7]. The use of CTAs is also useful for assessing GCA-induced complications such as arterial thrombosis, aortic aneurysms and dissections, and extracranial vascular abnormalities. In addition, our patient’s atypical stroke presentation necessitated an echocardiographic assessment to rule out secondary causes of stroke. Importantly, in view of the potential aortic-coronary involvements in GCA, all patients with GCA should have an echocardiogram, in addition to the above imaging modalities. A transthoracic echocardiogram provides an initial assessment tool for coronary artery vasculitis and other cardiac complications in patients with GCA and should be performed at presentation and upon follow-up post-treatment [9].

A systemic review and meta-analysis of cohort studies revealed that patients with GCA are at a 40% elevated risk of strokes [10]. Its relative risk is highest in the first month after the diagnosis of GCA [11]. Bilateral vertebral artery occlusion is seen as one of the rarest aetiologies of stroke and a highly fatal complication of GCA. Similar to our patient, in GCA-induced acute stroke, the vertebrobasilar territory is more commonly affected.

Our patient experienced visual disturbances prior to her admission. Visual loss is one of the most feared complications of GCA as it is irreversible and can be bilateral if not urgently treated with high-dose corticosteroids. Visual complications are reported in up to 30% of patients with GCA [2]. Visual loss is mainly caused by anterior ischaemic optic neuritis - ischaemia to the optic nerve - as a result of vasculitis involving the ophthalmic artery, branches of the posterior ciliary artery, and/or less commonly the retinal arterioles [12]. The visual loss could also be due to central retinal artery occlusion and vertebrobasilar stroke causing cortical blindness.

Both GCA and the intraventricular thrombus could have contributed to this patient’s visual loss, which was preceded by amaurosis fugax to the right eye and rapidly progressed to bilateral visual loss. Intraventricular thrombus could contribute to central retinal artery occlusion. Her pre-existing vascular risk factors also suggested an atherosclerotic origin to her presentations. This patient was found to have homonymous hemianopia with macular sparing during her first admission. It is worth noting that a posterior cerebral artery infarct is the most common cause of such presentation of cortical blindness. An echocardiogram demonstrated an intraventricular thrombus, which further presents diagnostic uncertainties in the case - whether all these presentations could be due to a cardiac embolus being thrown into the cerebral circulation, precipitating a cerebrovascular accident? It is proposed that both vasculitis and cardiac emboli could have contributed to the presentations seen in this patient, who also had some vascular risk factors for occlusive cerebrovascular disease. Her cortical blindness is presumably secondary to the stepwise progression of the MRI-proven bilateral occipital lobe infarctions in the vascular territory of the posterior cerebral arteries, which could have been driven by both vasculitis from GCA and the pre-existing cardiac emboli. More importantly, the presence of a coronary thrombus could also be due to the superimposed coronary vasculitis from GCA. Even though our patient did have a past medical history of hypertension and transient ischaemic attack, she developed bilateral posterior circulation infarcts and coronary thrombus without significant stenosis of the coronary arteries. This suggested that her presentations were likely due to complications of GCA rather than atherosclerosis. Since our patient had experienced a sub-acute onset of symptoms of GCA, this suggested that systemic vasculitis could be the most likely culprit of her delayed presentations.

The aetiology of GCA-induced stroke is much contested. The exact pathophysiology is unknown although several hypotheses have been proposed. It was initially attributed to atherosclerosis given the age groups and coexisting vascular risk factors in the affected patients. Recently, it was proposed that the presence of arterial inflammation may lead to endothelial damage and luminal irregularities that weaken the arterial wall, which can result in stenosis, dissection, and even aneurysms. Distal infarction or embolic occlusion may be secondary to dissection of the arterial wall or thrombosis. It is also important to recognise that ischaemic events and aortic aneurysms and dissections could be an initial, atypical manifestation of systemic GCA [3]. Furthermore, it is believed that the use of high-dose glucocorticoids may play a role in accelerating atherosclerosis and increasing vascular risks via inducing dyslipidaemia, hypertension, and hyperglycaemia [10].

Recently, there has been an increasing significance of an IL-6 inhibitor, tocilizumab, in the treatment of GCA, particularly in patients who are intolerant to corticosteroids. It is believed that GCA is associated with a marked elevation of IL-6, promoting a persistent inflammatory response in GCA. Tocilizumab, when combined with glucocorticoid therapy, has been found to increase remission rate and reduce total doses of corticosteroids over a year in a randomised control trial of 251 patients with GCA [13].

The incidence of GCA-induced vascular events is rare, let alone a case with complex multi-system aetiologies of presentations in a patient who was recently diagnosed with GCA. To the authors’ knowledge, our study could be the first described case of co-existing cerebral-coronary manifestations in a patient with biopsy-proven GCA presenting with bilateral visual loss in the literature. There are, however, a few published cases that describe coronary thrombosis and stroke secondary to GCA, with the majority demonstrating fatal clinical outcomes or poor prognosis including recurrence despite treatment with corticosteroids [1,14-17].

## Conclusions

Our report presented a clinically challenging and dynamic case that involved decision-making from multi-disciplinary teams. Our patient’s presentations may have been attributed to GCA, although atherosclerosis may also have a role. This case highlights the importance of detailed clinical assessments, biochemical investigations, and radiological studies in patients with suspected GCA, especially in a population otherwise susceptible to vascular events such as the elderly. In particular, we want to highlight to clinicians the use of imaging modalities including ultrasonography, contrast-enhanced body imaging, and echocardiography in patients with suspected or confirmed GCA. Temporary artery ultrasonography could be a useful, initial non-invasive assessment of GCA in highly suspected patients. Contrast-enhanced CT and MR imaging and echocardiograms are useful in assessing secondary thromboembolic disease and monitoring disease progression. A high index of suspicion is necessary and clinicians should be alert to the rare yet severe complications of GCA as early intervention with steroids may potentially improve prognosis.
